# *Rhizosolenia* mat diatoms associate with nitrogen-fixing microbes

**DOI:** 10.1093/ismeco/ycaf159

**Published:** 2025-09-15

**Authors:** Kendra Turk-Kubo, Mar Benavides, Matthew M Mills, Sarah R Smith

**Affiliations:** Ocean Sciences Department, University of California at Santa Cruz, Santa Cruz, CA, 95064, United States; National Oceanography Centre, European Way, Southampton, SO14 3ZH, United Kingdom; Aix Marseille Univ, Université de Toulon CNRS, IRD, MIO UM 110, 13288 Marseille, France; Turing Centre for Living Systems, Aix-Marseille University, 13009 Marseille, France; Department of Earth System Science, Stanford University, Stanford, CA, 94035,United States; Moss Landing Marine Laboratories, San José State University, Moss Landing, CA, 95039, United States; Microbial and Environmental Genomics, J. Craig Venter Institute, La Jolla, CA, 92037,United States

**Keywords:** *Rhizosolenia*, diatoms, diazotrophs, N_2_ fixation, *nifH*

## Abstract

Some *Rhizosolenia* diatoms living in oligotrophic marine ecosystems are known to form large, conspicuous mats and are thought to be sources of new nitrogen to surface waters via vertical migration to the nitracline where subsurface nitrate is accessed for growth. These vertically migrating *Rhizosolenia* mats are chronically under sampled, and both the diatom species comprising the mats and the associated microbiome have not been characterized using modern molecular techniques. Here we present the first DNA-based analysis of *Rhizosolenia* mats collected in the North Pacific Subtropical Gyre. Using sequencing of 18S rRNA and *nifH* genes (a proxy for N_2_ fixation capacity), we report on the molecular diversity of mat-forming *Rhizosolenia* species, which include two newly sequenced clades, and an assemblage of associated N_2_-fixing microorganisms that is distinct from the non-mat associated water column assemblage. Our findings advance knowledge of oligotrophic diatom diversity and challenge prevailing views of their nitrogen sources, suggesting these mats may obtain nitrogen through association-based N_2_ fixation. Further work is needed to understand the nature of these associations, and whether *Rhizosolenia* mat communities are a significant unrecognized source of N_2_-fixation-derived new nitrogen to the oligotrophic surface waters.

## Introduction

*Rhizosolenia* is a cosmopolitan diatom genus, observed across broad latitudinal gradients from tropical to subpolar waters, including inland seas [1]. In the subtropical oligotrophic oceans, some *Rhizosolenia* sp. are known to have intracellular heterocyst-forming, filamentous, N_2_-fixing cyanobacterial symbionts (*Richelia intracellularis*), while other *Rhizosolenia* species are known to form large aggregates (or *“*mats”) visible to the naked eye and comprised of multiple morphologically distinct *Rhizosolenia* sp. [2–4] and a microbiome that includes bacteria and ciliates [5]. *Rhizosolenia* mats contribute significantly to primary productivity and carbon export fluxes [6–9] and are thought to be sustained by nitrate assimilation at the nitracline accessed through vertical migration [10]. However, reports of N_2_ fixation in non-*Richelia*-bearing *Rhizosolenia* and of intracellular bacteria found within the diatoms suggest a symbiotic interaction between mat-forming species and N_2_-fixing microbes [3]. Subsequent efforts to measure N_2_ fixation using acetylene reduction assays have not substantiated these findings [4], though short incubation periods (30–45 min) may have missed the active N_2_ fixation period or signals may have been undetectable over background ethylene. Thus, the relative importance of nitrate assimilation vs. diazotrophy in supplying *Rhizosolenia* mats with nitrogen and the impact of mat diazotrophy on nitrogen biogeochemistry in the oligotrophic oceans remains unresolved.

**Figure 1:**
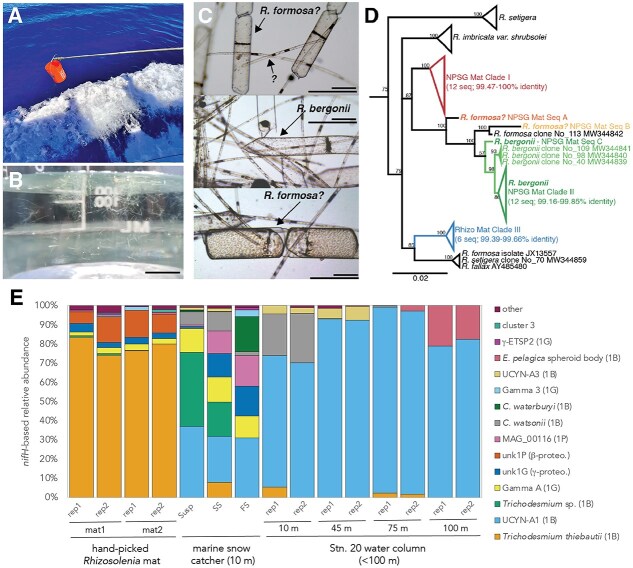
NPSG diatom mats contain diverse *Rhizosolenia* sp. and distinct diazotroph assemblages. (A) *Rhizosolenia* mats were bucket-sampled from the deck of the R/V kilo Moana (KM2206) at 27^o^ 13.3' N, 178^o^ 11.2′ E on June 19, 2022. (B) Mat samples in a jar (scale bar, 1 cm). (C) Light micrographs of different diatom mat species (scale bars, 50 μm, 100 μm). (D) Full-length 18S rRNA gene neighbor-joining consensus phylogenetic tree of NPSG mat sequences with reference *Rhizosolenia* sp. sequences from Medlin et al. [22] retrieved from the NCBI nucleotide database. All branches or collapsed clusters with color designate sequences retrieved from *Rhizosolenia* mats in this study. Branch labels show consensus support (%). (E) Relative abundances of diazotroph taxa based on *nifH* amplicon high throughput sequencing in mat, marine snow catcher and water column samples. Marine snow catcher deployments (10 m) captured three size fractions—Neutrally buoyant (or suspended), slow sinking, and fast sinking particles. Partial *nifH* fragments were amplified, sequenced and analyzed as described previously [23, 24] and in supplemental materials. MSC—Marine snow catcher; Susp—Suspended particles; SS—Slow sinking particles; FS—Fast sinking particles.

In June 2022, we encountered extensive fields of large *Rhizosolenia* mats (ca. 10s of cm in length) in the North Pacific Subtropical Gyre (NPSG). These mats remained visible to the naked eye throughout a 48 h station occupation situated in a frontal region between two eddies (Fig. S1). We gently collected the fragile mats with a bucket (Fig. 1A), and light microscopy revealed that they were a matrix of morphologically distinct *Rhizosolenia* species including large (>50 μm) and small (< 10 μm) diameter chains. These morphotypes were consistent with gross morphological descriptions of *Rhizosolenia castracanei*, *R. imbricata var. shrubsolei*, *R. formosa*, *R. debyana*, and *R. fallax* species reported in previously described multi-species mats (Fig. 1B and C) [3, 4]. None of the diatoms were observed to host the heterocyst-forming cyanobacterial symbiont *Richelia*, and quantitative PCR (qPCR) confirmed that *Rhizosolenia*-associated *Richelia* (Het-1) were rare in surface water at this station (6 × 10^2^  *nifH* copies L^−1^)*.* Although detailed characterization of diagnostic frustule features was not performed, precluding definitive morphology-based species identification, 18S rRNA gene analyses [11] indicated the presence of at least five distinct *Rhizosolenia* species or strains belonging to three major clades (Fig. 1D; Supplemental Material). Only one clade (NPSG Mat Clade II) and one additional sequence (NPSG Mat Seq C) showed sufficient similarity (>98%) to be reasonably assigned to *R. bergonii,* a species not typically associated with mat formation [12]. A single sequence (NPSG Mat Seq B) was identified as the mat-former *R. formosa,* and no sequences matched *R. imbricata var. shrubsolei* reference sequences. Two other major clades of NPSG mat-forming diatom sequences (Mat Clade I and Mat Clade III) could not be assigned to the species level due to limited reference sequence information for this genus. These sequences represent the first report of the molecular identities of confirmed mat-forming, non-*Richelia*-bearing, oligotrophic *Rhizosolenid* diatoms. Resolving the taxonomy of this group is particularly important because environmental sequence datasets (e.g. Ocean Barcode Atlas: [13]; CalCOFI: [14]; Northwest Atlantic: [15]) often classify *Rhizosolenia* only at the genus level, which is insufficient to constrain diatom mat biogeography underscoring the need to improve reference databases.

Diatom mats were associated with a diazotroph assemblage distinct from those in marine snow and ambient water (Fig. 1E). The diazotroph assemblage associated with *Rhizosolenia* mats was investigated using *nifH* amplicon sequencing from hand-picked mat samples and compared to particle and water column samples collected from the same site using a marine snow catcher (MSC) and Niskin-Conductivity, Temperature, and Depth (CTD) rosette, respectively (See Supplemental Material for methodological details). A non-cyanobacterial diazotroph (NCD) affiliated with a putative beta-proteobacterium (*nifH* cluster 1P) was specifically associated with the *Rhizosolenia* mats and not found in MSC or water column samples. Additionally, *Trichodesmium thiebautii* had high relative abundance within the mats, despite not often being observed in microscopic characterizations of the mats. Notably, the dominant mat-associated *Trichodesmium* sequences were distinct from MSC-associated *Trichodesmium*, another line of evidence supporting strain-specific mat associations (Fig. 1E). Collectively, these findings show that *Rhizosolenia* mats contain a distinct and varied community of both cyanobacterial diazotrophs (non-*Richelia*) and NCDs that does not simply reflect the diazotroph populations present in the MSC or water column samples, as would be expected if mats simply entrained the microbial populations present. Alternatively, mat assemblages may contain rare diazotroph taxa if the mat structure selects against the dominant diazotrophs in the water column or MSC samples. More work is needed to understand the variability of *Rhizosolenia* mat N_2_-fixing microbiomes, whether some mat-forming species host endosymbiotic NCDs as previously suggested [3].

Oligotrophic *Rhizosolenia* mat diatoms are thought to migrate to the nitracline to access subsurface nitrate to support their nitrogen demands [7]. However, uncertainty persists over whether some mat-forming species obtain nitrogen from diazotrophs. Reports of N_2_ fixation potential in *Rhizosolenia* mats have varied, perhaps since the specific *Rhizosolenia* species involved were not clearly identified, highlighting the importance of voucher specimens and a more precisely resolved *Rhizosolenia* phylogeny moving forward. However, our data provides evidence that mat-forming *Rhizosolenia* species, and possibly the associated mat microbiome, may obtain at least some of their nitrogen through a distinct assemblage of associated cyanobacterial diazotrophs and/or NCDs. If *Rhizosolenia* mats acquire nitrogen through N_2_ fixation, it represents a potentially significant and currently overlooked source of new nitrogen to the oligotrophic ocean. Current estimates indicate that N_2_ fixation in the oligotrophic waters of the North Pacific fuels ~50% of new production [16, 17]. Likewise, previously described associations between specific diatom hosts and heterocyst-forming cyanobacteria, including *Richelia intracellularis* associated with *Rhizosolenia*, and *Richelia euintracellularis* associated with *Hemiaulus* [18], are believed to support a significant portion of carbon export [16, 19]. Outside the predictable summertime export events, yearly N_2_ fixation is generally associated with smaller unicellular cyanobacterial diazotrophs (<10 μm; [20]). The most common approach for measuring N_2_ fixation uses small volume (~4 L) ^15^N_2_ tracer incubations of whole water, typically collected with Niskin bottles on a CTD rosette, and likely underestimates contributions from large and heterogeneously distributed diazotrophs (>20 μm; [21]). Thus, the magnitude of N_2_ fixed by heterogeneous and delicate *Rhizosolenia* mat communities likely remains uncaptured in current estimates, yet our work suggests N_2_ fixation within *Rhizosolenia* mats may add to diazotrophic support of carbon export. Determining if *Rhizosolenia* mats are an important and overlooked niche for diazotrophs and rely, even in part, on N_2_ fixation to supply their nitrogen demand has broad implications for prior estimates of their contribution to primary productivity and carbon export flux in oligotrophic ecosystems. Further work is necessary to clarify the nature of these associations using modern molecular techniques and directly measure *Rhizosolenia* mat associated N_2_ fixation. It is clearly time to revisit the old paradigm.

## Supplementary Material

RhizoDDA_MS_Suppl_Revision_Final_ENunlinked_ycaf159

## Data Availability

All *Rhizosolenia* 18S rRNA sequences are available in the nr database at NCBI Genbank under Accession numbers PV187244-PV187289. Raw *nifH* read data is available at NCBI SRA under BioProject # PRJNA1229115.
