# Supplementary material for: Rhizosolenia mat diatoms associate with nitrogen-fixing microbes
Source: ISME Commun. 2025 Sep 15;5(1):ycaf159. doi: 10.1093/ismeco/ycaf159 (PMC12499774; doi:10.1093/ismeco/ycaf159)
Supplement: RhizoDDA_MS_Suppl_Revision_Final_ENunlinked_ycaf159 [file rhizodda_ms_suppl_revision_final_enunlinked_ycaf159.docx]

**Supplemental Material**

***Rhizosolenia* mat diatoms associate with nitrogen-fixing microbes**

Kendra Turk-Kubo^1*^, Mar Benavides^2,3,4^, Matthew M. Mills^5^, Sarah R. Smith^6,7^

^1^Ocean Sciences Department, University of California at Santa Cruz, Santa Cruz, CA, U.S.A.

^2^National Oceanography Centre, European Way, Southampton, SO14 3ZH, United Kingdom

^3^Aix Marseille Univ, Université de Toulon, CNRS, IRD, MIO UM 110, 13288, Marseille, France

^4^Turing Centre for Living Systems, Aix-Marseille University, 13009 Marseille, France

^5^Department of Earth System Science, Stanford University, Stanford, CA, U.S.A.

^6^Moss Landing Marine Laboratories, San José State University, Moss Landing, CA, U.S.A.

^7^J. Craig Venter Institute, La Jolla, CA, U.S.A.

*Correspondence to [kturk@ucsc.edu](mailto:kturk@ucsc.edu)

**Supplemental Methods**

**Field Sampling**

Extensive fields of *Rhizosolenia* mats were visible from the deck of the of the R/V Kilo Moana (KM2206) throughout a 48h station occupation at the frontal region between two eddies (27^o^ 13.3' N, 178^o^ 11.2' E), where dynamic positioning was used to maintain station. *Rhizosolenia* mats for molecular analyses were sampled using a bucket affixed to a long pole from the deck at in the early afternoon (3:00 p.m.) on June 19, 2022. Water column samples were collected from discrete depths using a rosette of Niskin bottles (12 L) outfitted with a Seabird S*BE* CTD (Seabird Scientific, Bellevue, WA, USA) at 4:00 a.m. on June 19, 2022. Marine snow samples were collected using a 100 L Marine Snow catcher (MSC; OSIL, Havant, UK) deployed to 10 m on June 17, 2022 at 1:30 p.m.

**DNA sample collection**

*Rhizosolenia mats*

After bucket collection, portions of small *Rhizosolenia* mats (~1-2cm diameter) were gently transferred into clean petri dishes using wide-bore transfer pipettes. Mats were further sub-sampled (~0.5cm diameter size mat) for shipboard light microscopy (wet mount), and for DNA analysis. Samples for DNA were transferred into an acid-cleaned polypropylene syringe fitted with an acid-cleaned in-line 25mm polypropylene filter holder and gently filtered onto 25 mm diameter, 0.2 µm Supor® membranes (Cytiva, Marlborough, MA). Once complete, the membranes were transferred into 2 mL bead-beater tubes containing 1:1 mix of 0.1 mm and 0.5 mm glass beads (BioSpec, Bartlesville, OK), flash-frozen in liquid nitrogen, and stored at -80°C until DNA extraction.

*Marine Snow*

Suspended, fast sinking and slow sinking particles were subsampled from the MSC as described in detail by Reeder et al [1]. Briefly, following deployment to 10 m, the MSC device was kept shaded on deck and particles were allowed to settle undisturbed for 4 hours, following recommendations by Riley et al [2]. Suspended (Susp) and slow sinking (SS) fractions were then subsampled using acid-cleaned tubing into polycarbonate bottles. The fast sinking (FS) material (~300 mL) was pooled into a sterile 0.5 L polycarbonate bottle using serological pipettes.

*Bulk water column*

Samples for analysis of the bulk water column diazotroph assemblages were collected into 2L polycarbonate bottles (in replicate) that were pre-cleaned with >2h soaking in 2% trace-metal grade hydrochloric acid (Thermo Fisher Scientific, Waltham MA) the rinsed with MilliQ. For DNA samples, the 2L volume was filtered onto 25 mm diameter, 0.2 µm Supor® membranes (Cytiva) with peristaltic pumping with the acquired volume recorded, then transferred to bead-beater tubes and frozen as described above.

**DNA extraction**

*Rhizosolenia mats & MSC samples*

*Rhizosolenia* mat samples and MSC samples were extracted at sea using a quick DNA extraction protocol modified for easy use in the field based on the DNeasy Tissue Kit (Qiagen, Germantown, MD) [3]. This enabled the screening of several diazotroph taxa while at sea using quantitative PCR (qPCR) using a QuantStudio5 Real-Time PCR system (Applied Biosystems, Waltham, MA), which was our first hint that diazotrophs were present in *Rhizosolenia* mats and MSC-sampled particles (data not presented here). DNA extracts were stored at -80^o^C until processed for 18S rRNA gene and *nifH* amplicon analyses at UCSC.

*Bulk water column samples*

DNA and RNA was extracted with a AllPrep DNA/RNA Micro Kit (Qiagen). The protocol followed manufacturer guidelines, modifications described in Varaljay et al [4] that included agitating the samples for 2 minutes in bead-beater tubes in a 750 mL solution of RLT+β-mercaptoethanol (Sigma, Burlington, MA). Extracted RNA was stored immediately at -80°C while the DNA was tested for DNA content and purity with ND-1000 nanodrop spectrophotometer (Thermo Scientific, Burlington, MA) prior to being stored at -80°C.

**Community composition analyses**

*18S rRNA amplicon libraries & phylogenetic trees*

Full-length 18S rRNA gene sequences were amplified as described in Moon-van der Staay et al [5]. PCR reactions contained 2 µL of template DNA, Platinum™ Taq DNA polymerase (6 units; Invitrogen, Carlsbad, CA), 1X PCR Buffer (-MgCl_2_), 1.5 mM MgCl_2_, 250 µM dNTP mix, and 0.5 µM of primers MoonA and MoonB in a reaction brought up to 25 µL with RT-PCR grade water (Applied Biosystems, Waltham, MA, USA). To minimize contamination, all PCR reactions were carried out in an amplicon-free UV-hood as described by Turk et al [6], and no-template-controls were processed with each set of PCR reactions. Products were amplified using the following thermocycling parameters: 94°C for 5 min, followed by 30 cycles of 94°C for 1 min, annealing at 55°C for 2 min, and elongation at 72^o^C for 3 min. All samples were amplified in duplicate, screened for the correct products using gel electrophoresis, and pooled prior to cloning.

Amplicons were purified prior to cloning using the QIAquick Gel Extraction Kit (Qiagen) and cloned using the TOPO TA Cloning Kit for Sequencing (ThermoFisher, Waltham, MA) according to the manufacturer’s guidelines. Plasmids were purified from clones with recombinant plasmids prior to sequencing using the QIAwave Plasmid Miniprep Kit (Qiagen). Bi-directional Sanger sequencing of 18S rRNA gene inserts was carried out at the DNA Service Facility at the University of Illinois at Chicago.

Full-length sequences retrieved from the NCBI nucleotide database (nr) (using accessions and species assignments from [7] were aligned with NPSG mat sequences in Geneious Prime (2025.2.1) using the Super5 algorithm and MUSCLE 5.1. A Neighbor-Joining tree was constructed in Geneious Tree Builder (Genetic Distance Model: Tamura-Nei). The tree was resampled (100 times), and node labels show bootstrap values.

*Diazotroph assemblages via* nifH *amplicon next generation sequencing (NGS)*

Partial *nifH* fragments were amplified and sequenced as detailed in Turk-Kubo et al [8] and analyzed using the DADA2 ASV pipeline and workflow described in Morando et al [9]. Briefly, partial *nifH* gene sequences were amplified using a universal *nifH* nested PCR assay [10, 11], using reaction conditions and thermocycling parameters detailed in Turk-Kubo et al [8]. Barcoded libraries were prepared using a targeted amplicon sequencing approach was used to create as described in Green et al [12], using 5’ common sequence linkers [13] on the second round primers. Sequence libraries were prepared at the DNA Service Facility at the University of Illinois at Chicago, and multiplexed amplicons were bidirectionally sequenced (2 x 300 bp) using the Illumina MiSeq platform at the W.M. Keck Center for Comparative and Functional Genomics at the University of Illinois at Urbana-Champaign. Demultiplexed raw sequences are available under BioProject PRJNA1229115 in the Sequence Read Archive at NCBI.

Raw sequences were processed using a DADA2 pipeline [14] to identify amplicon sequence variants (ASVs) customized for the processing *nifH* fragments [9]. After processing raw data into ASVs, this pipeline is coupled with a series of post-pipeline stages that detect and discard spurious *nifH* sequences and annotate the subsequent quality-filtered *nifH* ASVs using multiple reference databases and classification approaches. This dataset was processed, and primary taxonomic IDs were determined using default parameters described in detail in Morando et al [9].

**Supplemental Figures**


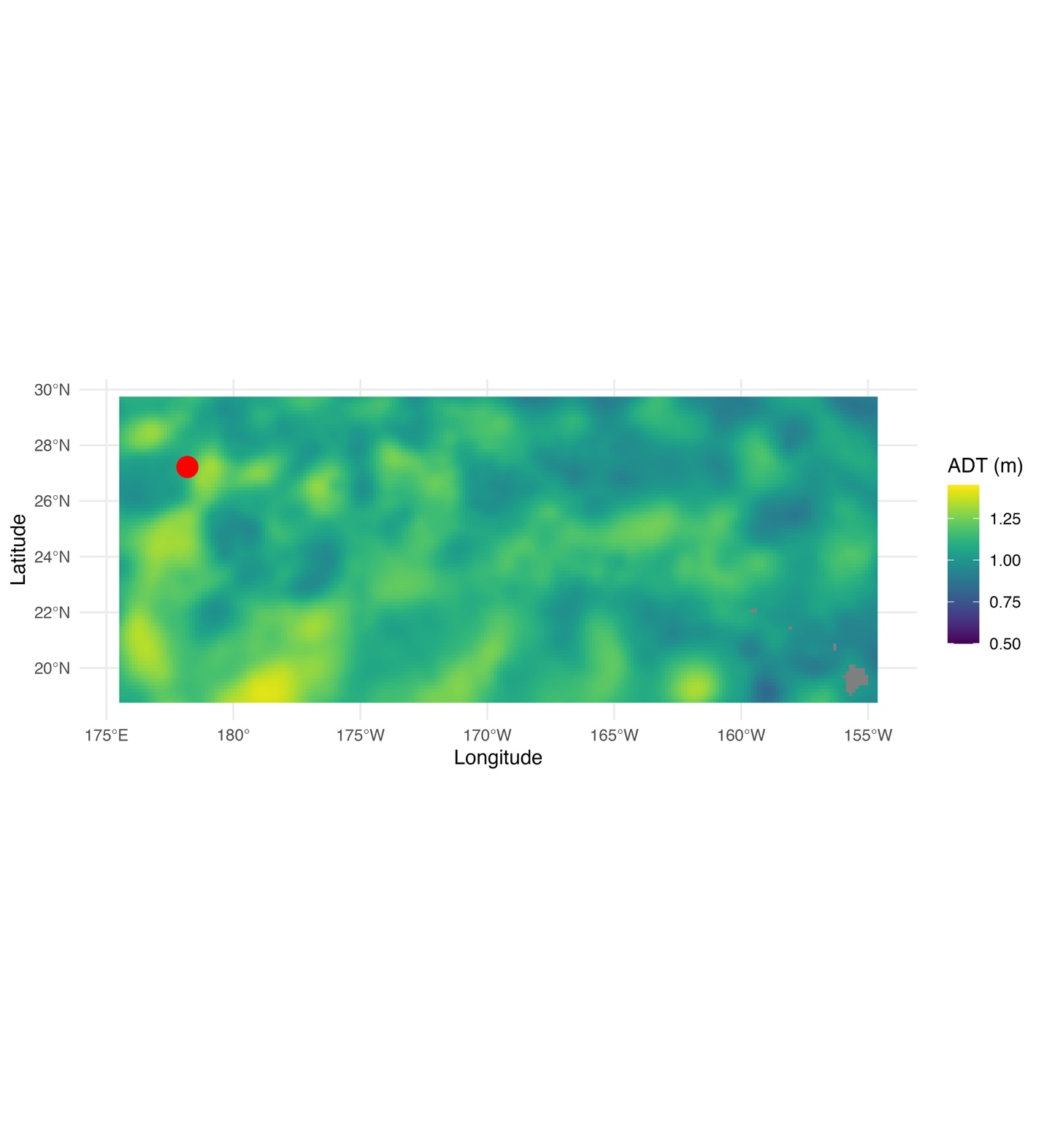


**Fig. S1. Absolute dynamic topography (ADT) – Station 20**. ADT composite for 20-21 June 2022, obtained from Copernicus Marine Data, processed by the DUACS multi-mission altimeter data processing system (<https://doi.org/10.48670/moi-00148>). The red circle indicates the position of the sampling station.

**Supplemental References**

1. Reeder CF, Filella A, Voznyuk A, Coet A, James RC, Rohrer T *et al.* Unveiling the contribution of particle-associated non-cyanobacterial diazotrophs to N_2_ fixation in the upper mesopelagic North Pacific Gyre. *Commun Biol*. 2025;**8**:287 https://doi.org/10.1038/s42003-025-07542-w

2. Riley JS, Sanders R, Marsay C, Le Moigne FA, Achterberg EP, Poulton AJ. The relative contribution of fast and slow sinking particles to ocean carbon export. *Global Biogeochem Cy*. 2012;**26**

3. Preston CM, Harris A, Ryan JP, Roman B, Marin III R, Jensen S *et al.* Underwater application of quantitative PCR on an ocean mooring *PloS ONE*. 2011;**6**:e22522 https://doi.org/10.1371/journal.pone.0022522

4. Varaljay VA, Robidart J, Preston CM, Gifford SM, Durham BP, Burns AS *et al.* Single-taxon field measurements of bacterial gene regulation controlling DMSP fate. *ISME J*. 2015;**9**:1677-86

5. Moon-van der Staay SY, De Wachter R, Vaulot D. Oceanic 18S rDNA sequences from picoplankton reveal unsuspected eukaryotic diversity. *Nature*. 2001;**409**:607-10

6. Turk KA, Rees AP, Zehr JP, Pereira N, Swift P, Shelley R *et al.* Nitrogen fixation and nitrogenase (*nifH*) expression in tropical waters of the eastern North Atlantic. *ISME J*. 2011;**5**:1201-12 https://doi.org/10.1038/ismej.2010.205

7. Medlin LK, Boonprakob A, Lundholm N, Moestrup Ø. On the morphology and phylogeny of the diatom species *Rhizosolenia* *setigera*: comparison of the type material to modern cultured strains, and a taxonomic revision. *Nova Hedwigia Beihefte*. 2021;**151**:223-47

8. Turk-Kubo KA, Henke BA, Gradoville MR, Magasin JD, Church MJ, Zehr JP. Seasonal and spatial patterns in diazotroph community composition at Station ALOHA. *Frontiers in Marine Science*. 2023;**10**:1130158

9. Morando M, Magasin J, Cheung S, Mills MM, Zehr JP, Turk-Kubo KA. Global biogeography of N_2_-fixing microbes: *nifH* amplicon database and analytics workflow. *Earth System Science Data Discussions*. 2024:pp.1-39

10. Zani S. Application of a nested reverse transcriptase polymerase chain reaction assay for the detection of *nifH* expression in Lake George, New York. *M S Thesis*. 1999;**Rensselaer Polytechnic Institute**

11. Zehr J, McReynolds L. Use of degenerate oligonucleotides for amplification of the *nifH* gene from the marine cyanobacterium *Trichodesmium thiebautii*. *Appl Environ Microbiol*. 1989;**55**:2522-26

12. Green SJ, Venkatramanan R, Naqib A. Deconstructing the polymerase chain reaction: Understanding and correcting bias associated with primer degeneracies and primer-template mismatches. *PLoS ONE*. 2015;**10**:e0128122 https://doi.org/doi:10.1371/journal.pone.0128122

13. Moonsamy PV, Williams T, Bonella P, Holcomb CL, Hoglund BN, Hillman G *et al.* High throughput HLA genotyping using 454 sequencing and the Fluidigm Access Array System for simplified amplicon library preparation. *Tissue Antigens*. 2013;**81**:141-9 https://doi.org/10.1111/tan.12071

14. Callahan BJ, McMurdie PJ, Rosen MJ, Han AW, Johnson AJ, Holmes SP. DADA2: High-resolution sample inference from Illumina amplicon data. *Nat Methods*. 2016;**13**:581-3 https://doi.org/10.1038/nmeth.3869
